# Risks of neonicotinoid insecticides to honeybees

**DOI:** 10.1002/etc.2527

**Published:** 2014-04-01

**Authors:** Anne Fairbrother, John Purdy, Troy Anderson, Richard Fell

**Affiliations:** † ExponentBellevue, Washington; ‡ Abacus Consulting Services, CampbellvilleOntario, Canada; § Department of Entomology and Fralin Life Science Institute, Virginia TechBlacksburg, Virginia, USA; ‖ Department of Entomology, Virginia TechBlacksburg, Virginia, USA

**Keywords:** Neonicotinoid, Honeybee, Risk assessment, Insecticide

## Abstract

The European honeybee, *Apis mellifera*, is an important pollinator of agricultural crops. Since 2006, when unexpectedly high colony losses were first reported, articles have proliferated in the popular press suggesting a range of possible causes and raising alarm over the general decline of bees. Suggested causes include pesticides, genetically modified crops, habitat fragmentation, and introduced diseases and parasites. Scientists have concluded that multiple factors in various combinations—including mites, fungi, viruses, and pesticides, as well as other factors such as reduction in forage, poor nutrition, and queen failure—are the most probable cause of elevated colony loss rates. Investigators and regulators continue to focus on the possible role that insecticides, particularly the neonicotinoids, may play in honeybee health. Neonicotinoid insecticides are insect neurotoxicants with desirable features such as broad-spectrum activity, low application rates, low mammalian toxicity, upward systemic movement in plants, and versatile application methods. Their distribution throughout the plant, including pollen, nectar, and guttation fluids, poses particular concern for exposure to pollinators. The authors describe how neonicotinoids interact with the nervous system of honeybees and affect individual honeybees in laboratory situations. Because honeybees are social insects, colony effects in semifield and field studies are discussed. The authors conclude with a review of current and proposed guidance in the United States and Europe for assessing the risks of pesticides to honeybees.

## Introduction

The European honey bee, *Apis mellifera*, plays an important role as a pollinator for major agricultural crops, pollinating $15 billion to $20 billion worth of crops in the United States alone and more than $200 billion worldwide 1. In fall 2006, unexpectedly high colony losses reported by 6 commercial beekeeping operations in the United States were investigated and attributed to “fall dwindle disease” 2. These operations lost 30% to 90% of their colonies in the fall, and some of the remaining colonies were considered too weak to survive overwintering 2. This new syndrome was dubbed “colony collapse disorder.” Colony collapse disorder is characterized by the sudden loss of a colony's adult worker bees, while the queen and live brood are present in surviving colonies. The latter part of the description often has been ignored when characterizing a colony as affected by colony collapse disorder, and the diagnosis can be difficult to apply because the entire colony may be dead at the time of inspection. No specific pathogen was identified in affected colonies, but the condition was considered distinct from previously known afflictions of beehives 2. After the initial report by vanEngelsdorp et al. 2, a colony collapse disorder working group was formed by the US Department of Agriculture (USDA), and a survey was conducted to investigate overwintering losses of bees 3. However, the survey was based on respondents' opinions; no baseline was established for comparison of losses prior to the survey, and no programs were in place to ensure proper diagnosis of colony collapse disorder or other honeybee diseases 3. Overriding economic factors, such as chronically low return on investment and cheap imported honey, were not considered but could have contributed to inadequate hive maintenance. Thus, the global occurrence of colony collapse disorder remains unproven.

**The European Honeybee (Apis mellifera)**

The European honeybee is a semidomesticated colonial bee species and only 1 of more than 20 000 bee species worldwide. It is native to Europe and Africa and was brought to North America in the early 1600s to provide a source of honey for the colonists. The honeybee is a social insect, living in large colonies where individual bees (queen, workers, and drones) are specialized to perform specific tasks. A colony is started by a group of bees (swarm) containing a queen, whose primary job for up to 5 yr is to lay eggs. Eggs hatch in about 3 d and produce larvae, which pupate after 5 d to 6 d. The pupal period is variable depending on the type of adult bee that will emerge, lasting 7 d to 14 d. The queen lays both fertilized and unfertilized eggs. The unfertilized eggs produce drones, whose sole purpose is to fertilize new queens. Adult drones live for about 40 d to 50 d. Fertilized eggs produce females, either worker bees or queens. The majority of individuals in a hive are the workers, which perform all of the activities necessary for the maintenance and survival of the colony. The newly emerged adult worker bees spend approximately 3 wk in the hive, feeding larvae, processing honey, standing guard duty, and providing air conditioning by flapping their wings. At about 21 d of age, worker bees start foraging for pollen, nectar, and water to bring back to the hive. They communicate the location of suitable flowers to other worker bees through an intricate “waggle dance” performed on the combs in the hive. Adult worker bees live for 30 d to 40 d during the summer months and up to 7 mo to 8 mo during the winter. New queens are produced before swarming (colony reproduction) or on loss or failure of the existing queen. However, the first queen to emerge generally kills the other potential queens, so only 1 new queen is produced for each colony. New queens leave the hive for mating when 5 d to 8 d of age, mating with up to 20 drones. Eggs are laid and hatch larvae throughout the spring and summer, but brood production stops by late fall, and the bees cease foraging until temperatures warm again in early spring. Honeybees do not hibernate and are active during the winter months, living off of stored honey and clustering to maintain temperatures at acceptable levels.

**Figure:**
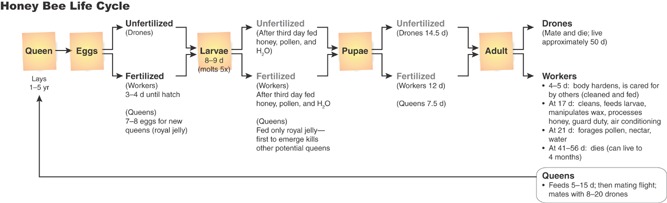

**Figure 1:**
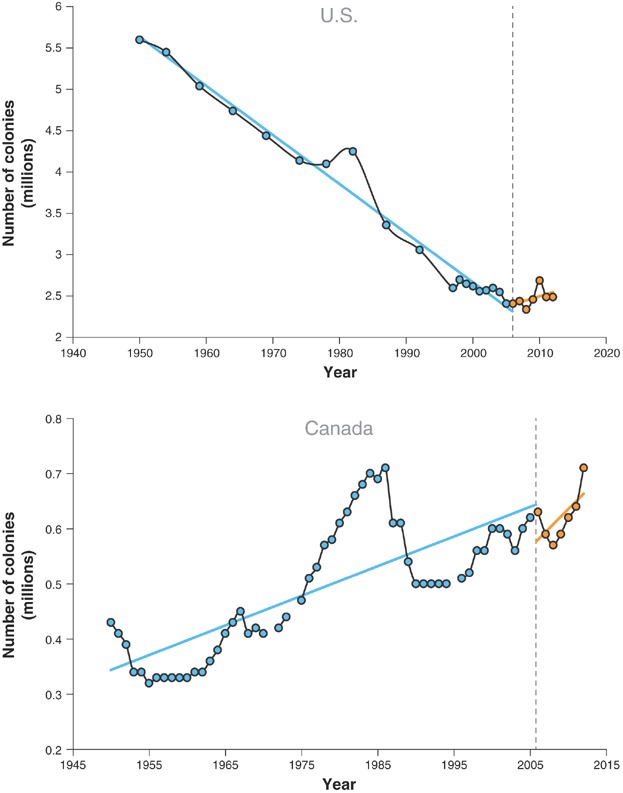
Number of managed colonies in the United States and Canada 1950 through 2012. Data adapted from Kluser et al. 4, USDA National Agricultural Statistics Service (www.nass.usda.gov), and Statistics Canada (www.statcan.gc.ca). Dashed line represents year when colony collapse disorder was first noticed (2006).

In fact, a report by the United Nations Environment Programme (UNEP) 4, among others, indicates that the number of managed colonies—bees kept in human-made hives managed for either honey production or pollination—is increasing worldwide, albeit with localized declines in some areas. In Europe, an overall decrease in managed honeybee colony numbers began in 1965 but was reversed in 2001, and numbers are now increasing. Records from the United States show a nearly constant rate of decline in the number of managed colonies since 1950, except for a slight increase in the late 1970s, followed by a steep decline after the arrival of the parasitic mite *Varroa destructor* in 1987 (Figure 1). As in the European Union, this trend reversed in 2005 when the number of managed colonies in the United States began to increase, in spite of higher overwintering losses. The long-term trend of managed colonies in Canada since 1950 is distinctly different from that in the United States (Figure 1). Aside from a decrease in the number of colonies following the arrival of *Varroa* mites in 1987, there has been an increasing trend from 1950 to the present. The number of managed colonies has shown a strong increase in Asia since 1991 and has also increased in Africa and Australia.

The primary basis for changes in the number of managed colonies in any country is economics 1. Historically, honeybees were raised for honey production, which continues to be the largest economic driver for beekeepers in Europe and Asia 5. In the United States, however, most hives in commercial operations are managed for pollination services. National Agricultural Statistics Services records show increasing income per hive since approximately 2005, driven by both increased acreage of crops that require pollination and the high demand for pollination of specialty crops such as almonds. Meanwhile, reduced subsidies for honey production by the USDA have reduced the interest in honey production. Today, more than 65% of commercial bee colonies in the United States are managed for pollination services 2.

The rate of colony losses as a result of weakening and mortality is a separate phenomenon from the trends in the number of managed colonies, but the 2 are often confused in both the media and technical reports. Since approximately 1998, more frequent colony losses have been reported in some regions of Europe, and similar increases in loss have occurred in the United States and Canada since 2006. Although details of colony loss rates are not available for Asia, there have been several episodes of major losses caused by mites and disease. These losses resulted in a series of reports in the scientific literature and news media that raised concern about populations of honeybees and pollinators in general 3. Annual losses are particularly evident in early spring when the colonies are inspected after overwintering, but the losses have not been uniform; they are highly variable in location and from year to year, showing no correlation with agricultural practices 4.

In contrast, Australia has not reported increased honeybee losses, and there have been no confirmed increases in colony losses from Africa 4. The issue of increased colony losses per year is complicated by the lack of a rigorous diagnostic and reporting network and changes in the definition of “colony loss” 4. A standard method for epidemiologic research has been issued by the International Bee Research Association to standardize and improve the quality of research in this area 6.

## Possible Causes of Colony Loss

**Figure 2:**
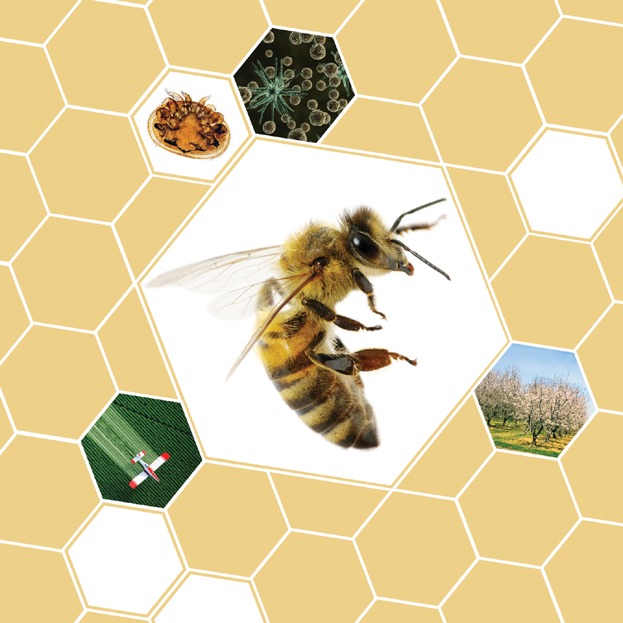
Among the many postulated causes of honeybee mortality are the varroa mite, multiple viruses, overwintering and pollination stress, and pesticide exposure.

Articles have proliferated in the popular press suggesting a range of possible causes for colony collapse disorder and the general decline of bees, such as mobile phones, pesticides, genetically modified crops, habitat fragmentation, and introduced bee diseases and parasites (Figure 2) 7. Poor bee husbandry, causing bees to go into winter in poor condition, has also been targeted as a possible cause of honeybee declines in the United States and Canada 5. In the United States, the demanding schedule of moving bees long distances for pollination is also a factor. More than half of all commercially managed honeybees in the country are brought to the almond orchards in the south Central Valley of California in early February, immediately after the overwintering period, and are left to forage on a single crop until all flowering is complete. Not only does this stress the colonies, but the aggregation of bees from around the country in a single locale creates a situation perfectly suited for exchange of disease and parasites. These colonies then disseminate around the country, where the bees are used to pollinate other crops such as apples in Washington and cranberries in Delaware, further increasing the possibility of spreading diseases and parasites. Additionally, some crops, particularly blueberries, do not provide adequate nutrition. Colony losses of 30% as a result of starvation are considered typical for these crops, compared with the average 22% loss that US beekeepers typically consider acceptable 2.

The Prevention of Colony Losses (COLOSS) research network, which now includes more than 300 scientists from 40 countries, was formed to understand and prevent large-scale losses of honeybee colonies. The COLOSS network developed a series of reviews of the large body of new research into causes of honeybee loss, including a special issue of the *Journal of Apiculture Research* in 2010 5. These scientists concluded that interactions among multiple factors are the most probable cause of increased colony losses, including the involvement of *Varroa* mites (*V. destructor*)—both alone and in combination with endemic and newly introduced viruses—the microsporidium fungi *Nosema apis* and *Nosema ceranae*, and pesticides and other factors such as forage losses and poor nutrition. Other reports excluded pesticides as a causal factor but agreed on the concept of multiple factors and the central role of *Varroa* mites and disease (e.g., Staveley et al. 8). These findings have been echoed by the UNEP, which concluded that the worldwide bee losses are not correlated to the use of pesticides and identified the *Varroa* mite as the primary concern, noting that “pathogens and pesticides are also being studied” 4. Using a causal analysis approach, Staveley and coworkers supported the UNEP conclusion that the major factor in reduced survival of bee colonies is *Varroa* mites; neonicotinoid pesticides were rated as unlikely to be the sole cause but could not be excluded as a contributing factor 8.

*Varroa destructor* is native to Asia, moving from its original host, the Asian honeybee (*Apis cerana*), when the European honeybee was brought to Asia. These mites have since been distributed around the world, except for Australia, through global commerce and the movement of honeybees, arriving in Europe and North America in the 1980s 1. Viral diseases of bees have been known for many years, and at least 8 of the 24 known viruses are transmitted by the *Varroa* mite. These mites are highly efficient vectors, transmitting viruses to brood or adult bees through the cuticle as well as through the normal routes of feeding or sex, and virus virulence increases during replication within the mite vector, resulting in death within days after inoculation 9. Dead and dying bees are removed from the hive, reducing the hive population sometimes quite dramatically. The visible symptoms of viral infection are unreliable, and multiple viruses may be present at the same time 6. New molecular techniques have shown that viruses may cause latent infections, living in the bees with no apparent signs of disease and suddenly transitioning to virulence, possibly caused by high mite populations 6. It has also been established that genes responsible for the immune response are downregulated during mite feeding 10, which may increase bee susceptibility to viral infection as the result of a compromised immune system.

The difficulty in correctly identifying the cause(s) of widespread bee mortality was illustrated by Neumann and Carreck 5, who have drawn parallels between the experiences with Isle of Wight disease that occurred in the United Kingdom more than 100 yr ago and the current issue with colony collapse disorder. Isle of Wight disease resulted in large-scale mortality, and there was a time when every colony lost in the United Kingdom was attributed to Isle of Wight disease; for a long time, the underlying cause was unknown. Many cases were misidentified as tracheal mites, and many still believe that to have been the cause, although recent studies have shown that Isle of Wight disease was actually a combined effect of acute bee paralysis virus, bad weather that inhibited foraging, and an excess of honeybee colonies compared with the amount of forage available 5. For most viruses, visual diagnosis of infection is unreliable. Symptoms in many cases are nonspecific, multiple viruses are usually present in a colony, and a definitive diagnosis is possible only through genetic analysis 6.

## Pesticides

While there is a growing consensus among scientists that the primary concerns for beekeepers are *Varroa* mites, nutrition, and diseases 8, many investigators and regulators have focused on the possible role that insecticides, particularly the systemic neonicotinoids, may be playing in bee health. Direct mortality from pesticides is considered to be limited to isolated incidents of exceptional circumstances 4,11. However, there remains a high level of concern among the public, many regulators, and some scientists, that pesticides may be responsible for weakening honeybees and making them more susceptible to disease, cold, or nutritional stress, or for affecting their learning ability 11.

Neonicotinoid insecticides are neurotoxicants and therefore have been of particular concern for sublethal effects in honeybees. This class of insecticides was considered a major milestone for integrated pest- and resistance-management programs at the time of their development, combining features such as broad-spectrum activity, low application rates, low mammalian toxicity, target specificity, upward systemic movement in plants, and versatile application methods. The neonicotinoid insecticides include imidacloprid, acetamiprid, clothianidin, thiamethoxam, thiacloprid, dinotefuran, nithiazine, and nitenpyram, which are marketed under a variety of trade names. Of particular concern for pollinators is their use as seed coatings, and the potential exposure that results from subsequent distribution of the insecticide throughout the plant, including pollen, nectar, and guttation fluids. There also is a concern for exposure to dust from treated seed in the exhaust air emitted by certain types of planters. In January 2013, the European Food Safety Agency (EFSA) released reports assessing the risks to honeybees of 3 neonicotinoid insecticides (imidacloprid, clothianidin, and thiamethoxam), explicitly acknowledging areas where data required to follow the current risk-assessment approach were unavailable 12. This was followed in April 2013 by the European Union placing a 2-yr moratorium on the use of neonicotinoid insecticides in an effort to reduce bee losses while the data needed for more accurate characterization of risks from these chemicals are developed. While sublethal effects of neonicotinoids on the foraging behavior of bees have been documented in many laboratory studies, similar effects have not been observed in field studies at field-realistic dosages 11, causing many regulators and scientists to question the applicability of the laboratory findings in a risk-assessment context. Not only are free foraging bees difficult to monitor but the measurement of quantifiable effects at the colony level from altered foraging behavior has not been possible. Consequently, the decision to suspend the use of these products was not unanimously supported by the European countries. The US Environmental Protection Agency (USEPA) has accelerated the schedule for registration review of the neonicotinoid insecticides and added a warning about honeybees and other pollinators to the labels of these products but, at the time of this writing, has declined to ban or suspend registration of these products.

A comprehensive review of more than 100 publications relating to neonicotinoids and honeybees has been published by Blacquiere et al. 11. We summarize the information that describes how neonicotinoids interact with the nervous system of honeybees and what is known about their effects on individual honeybees in laboratory situations. Because honeybees are social insects, the aggregate effect of the colony changes how individual bees are exposed and how they protect themselves from toxicants; therefore, we have included a discussion of what is known about neonicotinoid exposure in semifield and field conditions. We conclude with a review of the current and proposed methods for assessing the risks of pesticides to honeybees to highlight the evolution of our understanding of the interactions of chemicals and honeybees in commercial agriculture to increase crop production.

**Neonicotinoid Insecticides**

Neonicotinoid insecticides were developed in the 1990s as an alternative to more broad-spectrum pesticides. They are a synthetic form of nicotine, which is a naturally occurring insecticide, and target the same receptors in the insect nervous system. They are registered for use on a variety of crops and are effective against a wide range of sucking insects as well as chewing insects such as beetles and some Lepidoptera, particularly cutworms. Neonicotinoid insecticides can be applied as either a foliar spray or a seed coating. Labeling instructions specifically warn against applying the foliar spray during times that the plant is flowering. When seed is coated with the product, the plant incorporates the pesticide into its tissues as it emerges and grows, including transferring some of the product to pollen, nectar, and guttation fluid (guttation fluid is water that is transpired by the plant and forms small pools at the base of the leaves in early morning when humidity is high). To coat the seed, the insecticide and other substances (e.g., fungicides, nutrients, rhizobia) are mixed with various materials such as polymers, talc, and lime. Heating is used to anneal the coating to the seed. Seed coating is done in large, automated rotary coaters that can rapidly coat very large quantities of seed. There are 8 active ingredients registered in the United States, of which imidacloprid is the most widely used. The European Union instituted a 2-yr ban (2013–2015) on the use of these products while gathering additional information about their risks to polllinators.

Acetamiprid—Registered in 2002 for use on leafy and fruiting vegetables, cole crops, citrus, cotton, pome fruits, and ornamentals. Effective against ants, beetles, boxelder bugs, centipedes, chiggers, cockroaches, crickets, earwigs, firebrats, fleas, gnats, flies, millipedes, mosquitoes, moths, pillbugs, scorpions, silverfish, spidermites, spiders, stink bugs, ticks, termites, and wasps.

Clothianidin—Registered in 2003 for corn, canola, grapes, pome fruit, rice, tobacco, turf, and ornamentals. Effective against a wide variety of turf and sucking insects, including flies, beetles, moths, and true bugs.

Dinotefuran—Registered in 2012 for cotton, mustard, turf, lawn-and-garden use, vegetable crops, and residential indoors. Effective on a broad spectrum of insects, including aphids, whiteflies, thrips, leafhopper, leafminer, sawfly, mole cricket, white grubs, lacebugs, billbugs, beetles, mealybugs, sawfly larvae, and cockroaches.

Imidacloprid—Registered in 1992 for cotton, rice, cereals, peanuts, potatoes, vegetables, pome fruits, pecans, and turf. Effective against sucking and soil insects, whiteflies, termites, turf insects, and Colorado potato beetle.

Nitenpyram—Registered as a veterinary product for use on cats and dogs for flea control.

Nithiazine—Registered in 2011 for house fly control in animal facilities (poultry, feedlots, dairy, stables) and industrial locations.

Thiacloprid—Registered in 2003 for use on cotton and pome fruits. Effective against a variety of sucking insects, including aphids and whiteflies, codling moth, and plum curculio.

Thiamethoxam—Registered in 2001 for a wide range of vegetables, ornamentals, pome fruit, citrus, cotton, and rice. Effective against sucking and chewing insects, including aphids, thrips, beetles, centipedes, millipedes, sawflies, leaf miners, stem borers, and termites. Clothianidin is a transformation product of thiamethoxam.

### Neonicotinoid chemistry and modes of action

The commercialization of neonicotinoid insecticides is based on the structural improvement of nithiazin, an insecticidal nitromethylene molecule that targets insect nicotinic acetylcholine receptors (nAChRs), blocking the receptors and causing paralysis and death 13. The neonicotinoid insecticide imidacloprid (containing the nitroimine substructure) was the first commercially successful product in this class in the early 1990s, followed by clothianidin and thiamethoxam (containing the nitromethylene moiety) and then acetamiprid and thiacloprid (containing the cyanoimine moiety) in the early 2000s 14. The selectivity and action of neonicotinoids result from the interaction of nitrogen atoms in the imidazolidine ring with the amino acid residues of insect nAChRs 13. An open imidazolidine ring increases the efficacy of neonicotinoid insecticides toward insect cholinergic neurotransmission 14.

The honeybee nAChRs are Cys-loop ligand-gated ion channels that mediate fast synaptic transmission in the cholinergic nervous system. They are composed of 5 homologous α or non-α subunits arranged around a central ion channel and act as molecular switches in the cell membrane to control the influx of cellular cations (i.e., Na^+^, Ca^2+^, K^+^), which elicit neurotransmission 14. The honeybee genome sequence reveals 10 to 12 nAChR subunit–encoding genes that can exist as either homomers or heteromers of different α subunits or, more often, as combinations of α or non-α subunits 14,15. A series of amino acids has been identified that contribute to the selective interaction of the receptor with neonicotinoid insecticides 14. Diversity in the type of nAChRs across honeybee populations contributes to differential sensitivity to neonicotinoids and resilience of honeybees toward neonicotinoid insecticide exposure. The honeybee nAChR gene families are relatively small compared with those of other animals; posttranslational processes, however, including alternative splicing and mRNA editing, can produce protein products that exceed the number of nAChR genes and generate multiple subunit-encoding genes that increase the number of nAChRs 15. In addition, the honeybee possesses at least 1 highly divergent nAChR subunit that might be important for species-specific physiological function(s) 15. Major nAChR mutations have not been described in honeybees, but there are reports of “race-based” differences that may lead to insecticide sensitivity and resilience 16.

#### Neonicotinoid metabolism and detoxification

Insecticide metabolism has been given little consideration in most of the studies looking at chronic or sublethal effects of neonicotinoid insecticides on bees. The honeybee has a limited arsenal of detoxification proteins to withstand neonicotinoid insecticide exposures, and some authors have concluded that this makes them more sensitive and less able to develop tolerance to these pesticides 17. Although the capacity with which honeybees detoxify these chemicals is limited, honeybees have been shown to rapidly metabolize several of these compounds 18,19. Cresswell et al. 19 found that honeybees consuming imidacloprid in their diet in amounts as high as 2 ng/d for 8 d continuously eliminated the compound with no evidence of accumulation. This rapid metabolism and elimination of low doses of neonicotinoid insecticides raise questions about the reliability of conclusions of effects studies based on accumulated dosage levels and indicate that exposure to a single high dose is not equivalent to the same dose divided over time.

Despite the shortage of cytochrome P450 monooxygenases, esterases, and glutathione *S*-transferases in the honeybee, honeybees have a wide range of detoxification capacities for insecticides, which results in highly variable sensitivity or resilience to these chemicals 17. The haplodiploid system of the honeybee may contribute to reduced genetic diversity and to the limited detoxification proteins, although this has not yet been proven. Alternatively, it has been suggested that the highly social behavior of the honeybee is responsible for the lower number of detoxifying genes because these behaviors result in less exposure of reproductive individuals to environmental stressors, including pesticides 17.

### Effects of neonicotinoid exposure on honeybees

Because insects are the target organisms for use of neonicotinoid insecticides, and their use as systemic chemicals results in potential exposure via pollen, nectar, and guttation fluid, risks to honeybees and other pollinators has been a major focus of research in the last 15 yr 11. Both acute and chronic tests have been conducted in the laboratory on honeybees, as well as tests of sublethal changes such as behavior and learning, which are important to the overall health of honeybee colonies. Laboratory testing allows for greater control over environmental conditions and reduces the impact of extraneous factors. However, as discussed in more detail in the *Field and semifield tests* section below, caution is needed when attempting to extrapolate the results of laboratory studies to the colony level.

#### Acute toxicity

Laboratory tests follow standardized procedures 6. For contact testing, worker bees are anaesthetized with carbon dioxide and treated individually by topical application. For oral toxicity testing, groups of bees are food-deprived for several hours and then fed 10 µL to 20 µL of test solution per bee, either from group feeders or individually. Although standardized testing procedures should reduce intertest variability, however, reported median lethal dose (LD50) values for neonicotinoids vary over several orders of magnitude, depending on the compound, method of exposure, and variability among bees. For example, contact LD50 values for imidacloprid range from 7.8 ng/bee to more than 242 ng/bee; oral LD50 values range from 3.8 ng/bee to more than 81 ng/bee; no-observed-effect doses range from 1 ng/bee to 5 ng/bee (e.g., Cresswell 18). The problem is complicated further because sensitivity to insecticides can vary with the age of the test bees, subspecies, season, nutrition, physiological condition, and handling during testing (e.g., Cresswell 18). The resulting large variability increases the uncertainty in risk assessments and makes pesticide-management decisions difficult.

#### Gut microbiota

Several researchers have reported that exposure of laboratory honeybees to neonicotinoid pesticides increases their susceptibility to gut pathogens such as *N. ceranae* 20–23. Alaux et al. 21, for example, reported that imidacloprid exposure significantly weakened bees and led to higher mortality from *Nosema* infections; however, their data show only additive effects at low exposure levels (0.7 parts per billion [ppb] and 7 ppb) and no interactive effects. Potentiating effects were observed only at high imidacloprid concentrations (70 ppb), levels that are well above field exposure concentrations. Vidau et al. 22 found increased levels of mortality in *N. ceranae–*infected bees exposed to fipronil (1 ppb) or thiacloprid (5.1 parts per million [ppm]) but no correlation with changes in the detoxification capacity of infected bees (as measured by activity of either glutathione *S*-transferase or 7-ethoxycoumarin-*O*-deethylase enzymes). Pettis et al. 23 also reported increased *Nosema* infections in caged, newly emerged bees taken from colonies that had been fed either 5 ppb or 20 ppb imidacloprid in protein patties for 5 wk to 8 wk. When bees that had continued living in the experimental and control colonies were tested for *Nosema*, however, there was no relationship between the amount of *Nosema* infection and imidacloprid treatment. In a more recent study, Pettis et al. 24 found that the consumption of pollen containing neonicotinoid residues (acetamiprid, imidacloprid, and thiacloprid) was associated with a reduced risk of *Nosema* infection.

The studies by Pettis et al. 23,24 illustrate the difficulty in extrapolating laboratory effects to field conditions when investigating susceptibility to gut pathogens. Honeybees harbor a characteristic bacterial complex in the gut that plays an important role in nutrient processing, degradation of toxic compounds, and defending against pathogens. Newly emerged workers possess few bacteria, and their primary food, bee bread, does not provide an adequate source for the development of the normal gut bacterial community 25. The establishment of a normal microbiota requires contact with the colony and food exchange with older nestmates 25. The isolation of newly emerged workers in cages for testing may lead to increased susceptibility to pesticides and pathogens because of an impoverished gut microbiota. Differences in physiology, stress levels, and the bacterial complex of the gut may explain why the standard practice of collecting newly emerged workers from brood frames placed in incubators for use in laboratory pesticide tests may lead to misleading and/or inaccurate results.

#### Behavior, learning, and memory

Honeybees rely on intricate behaviors to locate flowers and return nectar and pollen to the colony. Additional complex behaviors occur within the colony for nurturing and raising young, as well as other labor activities. Therefore, because neonicotinoids are neurotoxicants, there has been considerable concern and research on how they may affect bee behavior, including learning and memory 26. Locomotor ability is critical to foraging success and is affected only by acetamiprid in older bees. Studies with imidacloprid, thiamethoxam, or acetamiprid and thiamethoxam (newly emerged bees) showed no effects, even at high exposure concentrations 18,26.

Conversely, laboratory studies have shown that neonicotinoid treatment can affect both learning and memory in honeybees. However, significant effects are seen only when treatment levels exceed what is typically found in field situations. For example, Decourtye et al. 27 found significant effects of imidacloprid on conditioned responses and medium-term memory at exposures above 12 ng/bee. Honeybees foraging on nectar at the highest reported measured imidacloprid concentrations, 10 ppb, would be expected to consume no more than 3.2 ng/d (assuming maximal sugar consumption of 128 mg and 10 foraging flights/d) 28. Such levels would not be expected to affect learning or memory and would be metabolized during the course of the day. However, Williamson and Wright 29 also used the proboscis extension reflex assay to examine the effects of imidacloprid on adult honeybees of foraging age. Caged bees were fed approximately 1.3 ng/bee to 13 ng/bee. Consumption at the highest level tested (13 ng/bee) affected the rate of learning as well as both short- and long-term memory, whereas the consumption of 1.3 ng of imidacloprid (field-realistic levels) reduced the rate of learning only in spaced conditioning trials. Furthermore, the high levels of mortality in that study's control group during toxicity testing (∼30%) raises questions about the health and stress levels of the bees during testing.

Effects on learning and memory have yet to be demonstrated under field conditions 30, further emphasizing the difficulties of extrapolating from laboratory studies to the field-level responses. Field trials with imidacloprid have shown delays in homing behavior and foraging behavior but only at food concentrations of 100 ppb 31, a level 50 times higher than average nectar concentrations. Similar effects on homing behavior were found with thiamethoxam but only at concentrations of 67 ppb 32. Clothianidin has also been shown to affect homing flight behavior at concentrations higher than expected from field exposure (>1.09 ng/bee) 33.

#### Physiology and stress

Although there currently are no standardized laboratory tests to study either chronic or sublethal effects of pesticides on honeybees, the maintenance of bees under laboratory conditions prior to and during experimentation is viewed as a means to provide better control of extraneous variables. However, the caging of adult honeybees, whether individually or in groups, may not provide an accurate indication of how pesticides affect behavior or colony health under natural conditions. Caging not only increases stress but also limits normal interactions and behaviors that can affect both development and physiology. Access to dietary protein, for example, plays a key role in adult worker development. Young workers feed on bee bread, which provides the proteins, lipids, vitamins, and minerals required for normal growth. Inadequate diets not only affect adult sensitivity to pesticides but also can affect development of the hypopharyngeal glands, the ovaries, the fat body, and immune responses, all factors that have been implicated as sublethal effects of neonicotinoid insecticide exposure 18. Despite this caution, results from laboratory studies often provide the basis for conclusions about chronic and sublethal effects of neonicotinoid insecticides on honeybees and are extrapolated by inference to field conditions.

#### Field and semifield tests

The variability of results in laboratory tests with individual bees makes it difficult to extrapolate to consequences under field conditions. A meta-analysis of 14 laboratory and semifield studies by Cresswell 18 suggested that dietary intake of imidacloprid at field-realistic levels could have sublethal effects and reduce honeybee performance by 6% to 20%. However, field studies by Maus et al. 20, Stadler et al. 34, and Creswell 18 demonstrated that, although neonicotinoids applied according to the label directions result in contamination of nectar and pollen, they do not cause acute toxic effects on foraging honeybees or significant health effects to colonies. Monitoring studies by Blacquiere et al. 11 and others conclude that residue concentrations in crops following application of neonicotinoids at recommended rates are too low to cause significant sublethal effects to honeybees. Cutler et al. 35 reviewed the Incident Reporting Program of the Canadian Pest Management Regulatory Agency and reported 110 incidents involving field mortality of bees in Canada since 2007, only 6 of which occurred prior to 2012. Although the neonicotinoids were suspected in a majority of incidents, including a large number in Ontario and Quebec in 2012, more than 90% of these were classified as “minor,” meaning that less than 10% of honeybees in the colony were affected; most of the “major” incidents were attributable to other insecticide classes. It is significant that in Alberta, Canada, where beekeepers rely most heavily on canola (oilseed rape) for honeybee foraging, colony numbers and productivity have increased steadily in the time since neonicotinoid canola seed treatments were introduced, and productivity per colony exceeds 100 kg honey/yr. It also has been shown that the use of clothianidin-treated seed of canola has no significant impact on honeybee colonies 36.

In a major study of honeybee losses in Germany 37, the main factors that had a statistically significant association with colony losses were “(i) high *Varroa* infestation level, (ii) infection with deformed wing virus … and acute bee paralysis virus … in autumn, (iii) queen age, and (iv) weakness of the colonies in autumn. No effects could be observed for *Nosema* sp. or pesticides.” As in Canada, there was no association of high colony losses with access to oil-seed rape. Neonicotinoids were approved for agricultural use in the United States in 1994, and their use worldwide has increased significantly since then, except in France where their use was banned in 1999. However, reduced rates of colony survival were not reported in the United States until 2006, more than 10 yr after their introduction, while the ban in France has not arrested the decline of colonies in that country. These observations provide strong evidence that neonicotinoid insecticides are not the sole, or even primary, cause of honeybee colony loss, although it is not possible to discount potential interactive effects of neonicotinoids and other stressors.

Nguyen et al. 38 studied 16 apiaries in a radius of 3000 m of maize fields (*Zea mays* L.) treated with imidacloprid. Mortality rates were higher in apiaries that had larger numbers of colonies, although this mortality was reduced when a larger surface area of the maize fields was treated. They concluded that this pesticide did not influence honeybee fitness. They also found that a large number of the samples from the hives that were tested for imidacloprid also contained acaricides that were either prohibited for use in beehives or ineffective against the *Varroa* mite. The authors concluded that, because the treatment methods used by the beekeepers were inadequate for mite control, the high mortality rates were more likely the result of mite infestations and that imidacloprid seed-treated maize had no measurable impact on honeybees.

Krupke et al. 39 measured concentrations of imidacloprid, clothianidin, thiamethoxam, and several herbicides in and around fields immediately after planting with neonicotinoid-coated maize seed. During planting, abrasion of treated seeds produces dust that contains residues of the chemicals applied to the seeds. Depending on the type of seed and planting equipment used, the amount of dust generated may be substantial. Large seeds, like maize, generate the most dust; and air seeders may exhaust significant amounts of dust under field conditions. Although planter-emitted dust is composed primarily of talc that is added as a dry lubricant to promote the flow of seed through the planter, it also contains insecticide residues to which bees may be exposed if the dust drifts onto flowering weeds along the field margins. While Krupke et al. 39 found no deposition of the dust onto the anthers of corn in fields adjacent to an experimental test field planted with treated seeds, they did report measurable amounts of neonicotinoids and other pesticides in field soils and in some pollen samples taken from hives placed along the field edge, as well as in flowering weeds and dead bees immediately adjacent to other fields that had been planted with treated seed. Although the highest seed residues were found in samples collected from the planter prior to planting, the authors concluded that the dust generated from planting was sufficiently toxic to kill a large number of bees. However, Sgolastra et al. 40 showed that insecticide-induced mortality in forager bees at levels up to 10 times that of nontreated controls for 2 d to 3 d after sowing had no lasting effect on colony strength (number of adult bees and brood extension) or sociophysiological status (thermoregulation capacity, comb construction capacity). This study clearly demonstrated that the loss of a large number of individual honeybees can be tolerated by a colony during spring and summer. Mortality of forager bees is a frequent and natural occurrence during this time, which coincides with spring and summer planting; but colony losses occur primarily in the fall and during overwintering 40. The study also emphasizes the need to go beyond laboratory experiments on individual bees that can be used to determine the toxicity and mode of action of a pesticide, to semifield and field studies involving entire colonies prior to making a determination of risk.

## Risk Assessment Frameworks

Historically, potential risks to honeybees from pesticide applications have been assessed using 2 acute tests in initial tier studies: topical application for contact toxicity and feeding on a treated sugar solution for oral toxicity 6. An additional standardized test has been used for determining how long foliar residues remain toxic 6. All 3 of these tests investigate toxicity to individual honeybees. Chronic effects to bees are assessed only in higher-tiered studies that may be required on a case-by-case basis, involving exposures to spray applications in a simulated field environment (semifield study) or to field-applied pesticides (field study). As a result of the increasing concern over honeybee colony deaths and claims of a decrease in numbers of colonies in production, regulatory agencies have begun to look for risk-assessment frameworks with more realistic exposure scenarios to all life stages and castes in the colony, as well as relevance to other pollinator species. A SETAC Pellston Workshop held in 2011 established protection goals and proposed a tiered approach to assessing risks to pollinators 41. Subsequently, the USEPA convened a science advisory panel to review a proposed risk-assessment framework for bees and issued a white paper 42, and the EFSA appointed a panel of experts to develop a detailed approach for assessing risks to honeybees, resulting in the recent adoption of new guidance for the European Union 43.

### Tiered risk-assessment frameworks

The protection goals described by the Pellston workshop 41 and the USEPA 42 are protection of pollination services, honey production and other hive products, and biodiversity (i.e., protection of wild bumblebees and other pollinators). Goals of the risk assessment include assessing the effects of pesticides on colony strength (population size and demographics) and colony survival (persistence). The EFSA's risk-assessment goals 43 are similar; that is, to protect colony strength by protecting survival, reproduction, and development of colonies, as well as behavior of larvae and adult bees. The EFSA 43 operationally defines “colony strength” as the number of honeybees a colony contains and has determined that the magnitude of effects on colonies should not exceed 7% reduction in colony size. Additionally, mortality of forager bees should not be increased more than 1.5-fold compared with controls over a 6-d time period, or a factor of 2 for 3 d, or a factor of 3 for 2 d. Because the protection goal is at the colony level, it is particularly important for the emergent properties associated with the colony behaviors to be taken into account. For example, foraging bees exposed to neurotoxic pesticides may not return to the hive or may improperly communicate the location of forage plants. If this occurs, other worker bees will not know the location of the contaminated plants, so the probability of exposure is greatly reduced.

All of the proposed risk-assessment frameworks follow a tiered approach, with initial toxicity tests conducted by exposing individual honeybees in the laboratory, followed by more realistic exposures of whole colonies under semifield conditions (or “tunnel” studies) and large-scale field tests. This approach is intended to first assess the hazard of a chemical to bees—that is, assess the potential for the chemical to cause a toxic response without explicit consideration of exposure scenarios. The more comprehensive studies or models assess the *probability* of encountering real-world exposures. These higher-tier studies are particularly complex because individual bees interact within the colony in a manner that can either increase or decrease the colony's exposure, and the severity of the response can differ across castes and life stages. The EFSA guidance 43 acknowledges a level of conservatism because the highest tier tested is at the field—and not at the landscape—level, where honeybees may forage freely on exposed and nonexposed crops or weeds along field edges. Furthermore, the EFSA 43 specifically states that the assessment is conducted using a worst-case exposure scenario (e.g., 90th percentile of possible exposure values), to ensure protection of pollinators in over 90% of all treated fields.

### Exposure

Exposure pathways from foliar sprays include external contact and diet. Dietary exposures also occur from products incorporated into plant tissues through seed treatments or, occasionally, direct injection into the plant. Contact to foliar products can include residue on plants and dust from treated seeds during planting. For contact exposure estimates of foliar products, standard methods for determining residues on plant leaves as a function of concentration, spray volume, nozzle size, and drift can be used (e.g., USEPA's T-REX model 42); and results can be compared directly to the contact LD50 or other test end points.

**Figure 3:**
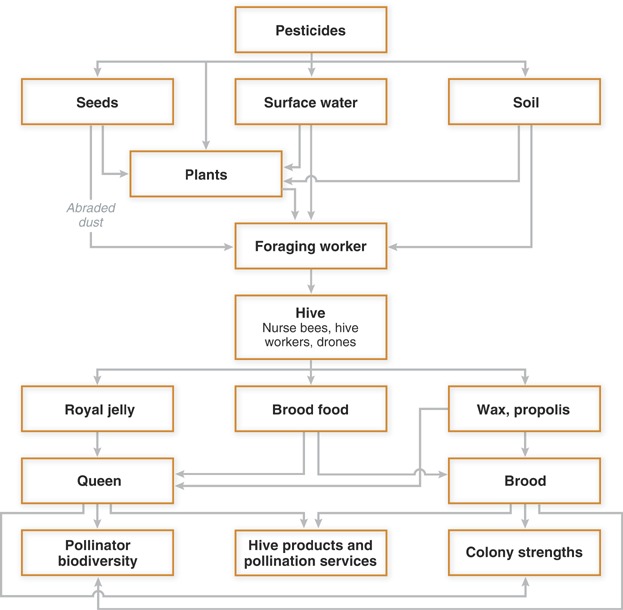
Conceptual model for bee risk assessment. Adapted from Fischer and Moriarty 41 and US Environmental Protection Agency 42.

Adult honeybees potentially can be exposed to systemic pesticides from several sources: seed coatings or soil, or through pollen, nectar, or guttation fluid (Figure 3). Brood and queens are exposed via processed pollen (brood food or royal jelly, respectively) and processed nectar (i.e., honey). Because different plant species take up and distribute chemicals differently, it can be difficult to accurately model concentrations in pollen, nectar, or guttation fluid based solely on application rate. Therefore, default values for dietary concentrations (i.e., pollen, nectar, and guttation fluid) have been proposed for tier 1 screening studies. Fischer and Moriarty 41 proposed adopting a default value of 1 mg/kg for systemic compounds and noted the lack of any information for foliar-applied products. The EFSA 43 also uses the 1-mg/kg default value for systemic compounds but additionally provides a set of default values for foliar applications, depending on whether the assessment is for exposures up- or downwind from the application event. These values are 7.6 mg/kg (downwind) to 10.6 mg/kg (upwind) for adults and 4.4 mg/kg (downwind) to 6.1 mg/kg (upwind) for larvae. Exposure values can be refined through measurements of pollen and nectar during semifield studies (see below). Pesticide concentrations in guttation water are estimated according to EFSA 43 based on their water solubility (100% soluble in the screening assessment, with reduced solubility estimates in the higher-tier assessments).

For higher-tier risk assessments of foliar pesticides (including dust from seed drilling), exposure is expressed as the total exposure from foraging on the crop, weeds within the field, weeds at the field margin, and adjacent crops. Carryover to subsequent years may also be examined. Thus, the relative proportion of the applied chemical that is in the various plant types needs to be calculated. In addition, the proportion of time that bees spend foraging on each plant type is included in the exposure estimate for all types of applications, including systemic products. These parameters can result in large uncertainties in the final exposure estimate, particularly if the pesticide itself may alter foraging behaviors. The time of day that bees are foraging can also alter exposure (e.g., guttation fluid is present only in the early morning when it is still too cool for foraging activity) 42. Therefore, for chemicals with high intrinsic toxicity, field studies would be needed to measure concentrations in and on the plants, pollen, nectar, and other hive products and to look for compensatory changes in honeybee behaviors.

### Effects

At present, the only end point with standardized protocols is mortality, although behavior is included in a semiqualitative manner. There are 2 standard protocols for quantifying pesticide-induced mortality: the contact and oral toxicity tests. Both are short-term studies, at 48 h and 72 h, respectively. Tests are conducted on individual worker bees at the cleaning/feeding stage of their life cycle. It has been proposed that these tests be extended to 96 h or 10 d for chronic exposure assessment (e.g., immobilization, incoordination, and hypo- or hyperresponsiveness), although additional research is needed to develop repeatable protocols 43. Additional tests for sublethal effects have been proposed, such as neuropathy (with the proboscis extension reflex test), learning behavior (with a maze test) 41, and effects on hypopharyngeal glands 43. Tests for additional life stages are also available and have been proposed for situations in which exposure via pollen and nectar is considered 43. This includes a honeybee first-instar larva dietary toxicity test being prepared as an Organisation for Economic Co-operation and Development standard test, in which survival, prepupal weight, duration of development, adult morphology, and behavior are recorded 6. Effects on bee brood can be investigated with colony exposure to pesticide in sugar solutions and monitoring brood mortality at 7 d and just prior to emergence, as well as pupal deformities just prior to emergence. However, these test methods have not yet been standardized 43.

Semifield studies where entire colonies are exposed to the crop or a mix of crop and weeds, either in cages, tunnels, or tents, have been standardized 6 as a means of assessing changes in behaviors under worst-case scenarios. The EFSA 43 recommends increased replication when conducting these tests to enhance statistical sensitivity. The use of a bee-attractive crop such as oilseed rape (*Brassica napus*), mustard (*Sinapis hirta*), or buckwheat (family Polygonaceae) is recommended to ensure high-percentile exposure and allow sufficient quantification of the pesticide in pollen and honey 41. Along with mortality at the front of the hive, behavioral end points include flight activity, foraging behavior on the treated crop (e.g., repellency), ability to return to the hive, and other behavioral abnormalities. Accurately measuring in-hive exposure (beyond concentrations in honey and royal jelly) can be difficult. Full field studies are rarely conducted. The primary assessment end points of field studies are forager returns and mortality, colony strength (number of bees), hive weight, overwintering success, presence of the same queen, and honey production. Secondary-effect end points are behavioral abnormalities, including behavior of foragers on flowers and of guard bees at the colony entrance, and disease resistance 6,41.

### Risk characterization

There is no harmonized approach to a tiered risk assessment to bees, although there is general agreement that early tiers should be more conservative, to minimize the potential for false negatives, and higher tiers should become more realistic. Current and proposed schemes begin with the study of effects to individual bees exposed to high pesticide concentrations under worst-case conditions and conclude with long-term, full-scale field assessments that are more reflective of realistic exposure levels. However, there is disagreement on whether behavioral end points (e.g., proboscis extension reflex) can be included in a standardized test because of the lack of guidance on interpreting ecological significance at various levels of effect. The USEPA 42 states, “Until there are sufficient data to establish plausible adverse outcome pathways with consistent and reproducible linkages between molecular initiating events and key events across multiple levels of biological organization to an adverse effect at the whole organism/colony/population level, it is difficult to make use of sublethal effects other than in qualitatively describing potential adverse effects.”

There is no agreement on what triggers a conclusion of unacceptable risk at the tier 1 screening level or whether to include assessment factors to account for uncertainties in the test results, extrapolations from laboratory to field, and interspecies differences. For chemicals that do not pass the tier 1 screen, the risk-management approaches suggest putting mitigation measures into place to reduce exposure below the screening level or conducting higher-tier studies to develop more accurate exposure metrics and to measure colony-level end points. Bee colonies have emergent properties that make it very difficult to extrapolate colony-level exposures from honey and bee bread and colony-level responses to field applications of chemicals.

## Conclusions and Summary

While it is undeniable that overwintering losses of commercial honeybee colonies are higher than they were in the recent past, there is no clear indication that pesticides are the root cause of such losses. The USDA survey shed light on the pattern of honeybee losses across the United States and concluded that such losses were unrelated to the patterns of agricultural pesticide use, in general, or neonicotinoid use, in particular. While beekeepers may have difficulty diagnosing a new phenomenon such as colony collapse disorder, they are familiar with other causes of colony loss; and pesticides ranked 8th on the list of possible causes of colony loss in the USDA survey 3. Additionally, the epidemiological evidence from Europe shows no correlation of honeybee losses to pesticide use and indicates the presence of causal factors other than pesticides, although it is not yet possible to completely discount potential interactive effects of neonicotinoids with other stressors. Finally, the time of year when increased mortality of honeybees is the late fall and over the winter, whereas the highest pesticide use occurs in the spring and early summer. The life span of forager bees is very short (approximately 1 mo), so the bees that may be exposed to the insecticide in the spring and early summer are not the same bees that overwinter in the hive. Additionally, it has been shown that neonicotinoids do not accumulate over time in the environment, the colony, or the honeybees. Given these 2 attributes of neonicotinoids and bees, it is not possible for the chemicals to have latent effects that are expressed months after application.

All of the neonicotinoid insecticides have been reviewed and approved in many jurisdictions around the world, including Europe, Australia, Japan, Canada, and the United States; and they have been used for more than 15 yr on a variety of crops. Therefore, a significant body of data from both laboratory and field studies is available to assess the risks to colonies of honeybees. The available data indicate that there may be effects to individual honeybees housed under laboratory conditions and exposed to unrealistically high concentrations of the insecticides. However, under field conditions and exposure levels, similar effects on honeybee colonies have not been documented. It is not reasonable, therefore, to conclude that crop-applied pesticides in general, or neonicotinoids in particular, are a major risk factor for honeybee colonies, given the current approved uses and beekeeping practices 1,3,4.

As the commercial honeybee industry evolves toward a pollinator-based economy, with fewer but larger companies and greater aggregations of bees, beekeepers are learning to manage their bees in a manner similar to that of other livestock operators. Herd (or colony) health depends on an in-depth understanding of animal nutrition and disease management, including routine treatment for parasites and surveillance for new, emerging foreign-animal diseases. Public awareness and scientifically sound studies funded by governments and agricultural interests, including agricultural chemical companies, have identified the interaction of multiple stressors, including parasites (*Varroa* mites), pathogens (viruses, *Nosema* fungus), and nutrition (monofloral vs polyfloral pollen and nectar resources), as primary factors influencing honeybee health. Sublethal effects of pesticides on behavior, learning, and immunity are subtle and may not be measurable at realistic exposure concentrations. The more robust risk-assessment frameworks being proposed and recently adopted in Europe provide guidance for a better initial analysis of possible effects of pesticides, but higher-tier assessments must be implemented to determine the realistically probable consequences of chemical use under field conditions. Assessing risks only under worst-case conditions with individual honeybees, divorced from properties provided by colony interactions, serves only to understand potential mechanisms of action of different chemicals but not their aggregate risks. Because both pesticides and pollinators are critical to the continuing success of worldwide agriculture, it is imperative that we learn to accurately and honestly assess the benefits and risks of their interactions on commercial honeybees and other pollinators.
